# Influence of Maternal Metabolic Status and Diet during the Perinatal Period on the Metabolic Programming by Leptin Ingested during the Suckling Period in Rats

**DOI:** 10.3390/nu15030570

**Published:** 2023-01-21

**Authors:** Pedro Castillo, Catalina Amadora Pomar, Andreu Palou, Mariona Palou, Catalina Picó

**Affiliations:** 1 Laboratory of Molecular Biology, Nutrition and Biotechnology (Group of Nutrigenomics, Biomarkers and Risk Evaluation), University of the Balearic Islands, 07122 Palma, Spain; 2 Health Research Institute of the Balearic Islands IdISBa, 07010 Palma, Spain; 3 CIBER de Fisiopatología de la Obesidad y Nutrición (CIBEROBN), 28029 Madrid, Spain

**Keywords:** lactation, leptin, metabolic programming, Western diet

## Abstract

We aimed to analyze the long-term metabolic effects of leptin supplementation at physiological doses during suckling in the offspring of diet-induced obese rats, together with the potential benefits of improving maternal diet during lactation. Thus, the offspring of: dams fed standard-diet (SD) (CON-dams), dams fed western-diet (WD) before and during gestation and lactation (WD-dams), and dams fed as WD-dams but moved to SD during lactation (REV-dams) were supplemented throughout suckling with leptin or vehicle, and fed SD or WD from weaning to four months. Under SD, leptin treatment significantly improved metabolic profile and body fat accumulation, with stronger effects in the male offspring of CON-dams and REV-dams. Under WD, the offspring of WD-dams presented metabolic alterations that were not evident in the offspring of REV-dams. Moreover, leptin supplementation improved glucose homeostasis in the male offspring of REV-dams. Conversely, leptin supplementation in females born to WD-dams and fed WD from weaning resulted in impaired insulin sensitivity and increased hepatic lipid content. These results highlight the importance of a balanced maternal diet during the perinatal period, especially lactation, for the subsequent metabolic health of the offspring and for the beneficial effects of leptin supplementation during suckling, more evident in the male offspring.

## 1. Introduction

The nutritional environment during prenatal and early postnatal life highly influences later metabolic health. Exposure to adverse conditions during these periods can negatively affect the proper early growth and development, thus increasing the predisposition to develop chronic diseases in adulthood [1,2]. In this context, there is wide evidence showing that maternal obesity and/or consumption of an obesogenic diet during lactation increases the risk of the offspring suffering from obesity-related comorbidities, although it is difficult to discern the contribution of both factors [3,4,5]. Of note, maternal intake of a westernized diet throughout gestation and lactation, without suffering from obesity previously to gestation, has been described as being sufficient to trigger metabolic disorders in the offspring [6,7,8]. In addition, we have recently shown that implementing a healthy diet during lactation in diet-induced obese rats attenuates the detrimental impact of Western diet (WD) consumption before and during gestation on phenotypic traits and circulating parameters of the offspring at weaning [9,10], although the longer-term effects are not known. 

Breast milk is considered the optimal nutrition for infant growth and development in early postnatal life [11]. In fact, breastfeeding, compared to infant formula feeding, has been linked with a lower propensity of the progeny to suffer from health disorders, both in the short and long term [12]. Besides its macronutrient composition, different bioactive compounds that are present in breast milk, including endogenously synthesized peptide hormones that distinguish it from infant formula, have been related to the health benefits of breastfeeding [13,14]. Among them, leptin is the most studied compound and for which there is more evidence on its critical role in the programming of future body weight and metabolic health [13]. In animal models, supplementation with physiological doses of leptin during the suckling period in offspring of normal-weight dams has been shown to improve metabolic health and prevent the development of obesity and related disorders later in life [15,16,17]. Moreover, leptin supplementation has been shown to greatly ameliorate the dysmetabolic phenotype associated with moderate gestational calorie restriction and exacerbated by WD exposure in adulthood [18,19,20]. In humans, there is also indirect evidence for the role of leptin during lactation, since a negative association between breast milk leptin levels and infant body weight has been reported by independent studies, particularly in those conducted with normal-weight women, although such a relationship is not as clear in overweight/obese women [13]. In fact, despite the essential role of leptin during lactation, the precise requirements of leptin and whether its beneficial effects during this critical window might depend on particular maternal conditions, such as obesity or exposure to unbalanced/obesogenic diets, are still open questions. This is of interest considering the important increase in the prevalence of overweight and obesity among women of reproductive age globally, particularly in low-middle income countries [21]. Therefore, here we aimed to assess whether leptin supplementation throughout the suckling period in the offspring of diet-induced obese rats exposed or not to WD conditions during lactation is capable of ameliorating the negative impact of such maternal condition on their metabolic health in adulthood, together with the potential benefits of improving maternal diet during lactation.

## 2. Materials and Methods

### 2.1. Animals and Experimental Design

The Bioethical Committee of the University of the Balearic Islands reviewed and approved the animal protocol (Exp. 2018/13/AEXP, 23 January 2019). Guidelines for the use and care of laboratory animals of the University were followed.

**Figure 1:**
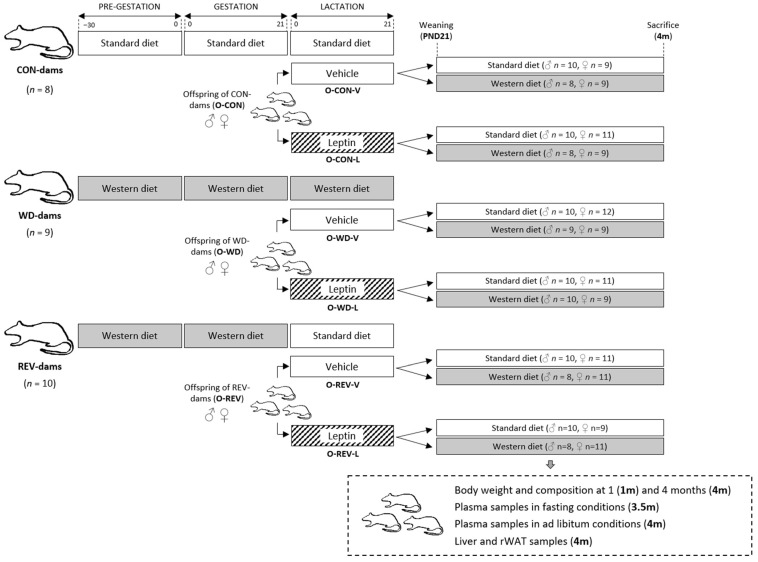
Representative scheme of the experimental design. Abbreviations: control dams (CON-dams), western diet dams (WD-dams), reversion dams (REV-dams), offspring of CON-dams (O-CON), offspring of WD-dams (O-WD), offspring of REV-dams (O-REV), vehicle (V), leptin (L), retroperitoneal white adipose tissue (rWAT).

A scheme of the experimental design described below is detailed in Figure 1. As previously described [9,10], virgin female Wistar rats were housed under controlled conditions (22 °C, a 12 h light–dark period and free access to food and water) and divided into two groups: dams fed with a standard chow diet (SD; 3.3 kcal·g^−^^1^, with 72.4% from carbohydrates, 8.4% calories from fat and 19.3% from protein; Safe, Augy, France) (*n* = 8) or with a high-fat and high-sucrose diet (western diet, WD; 4.7 kcal·g^−^^1^, with 43.0% from carbohydrates, 40.0% calories from fat and 17.0% from proteins; Research Diets, New Brunswick, NJ, USA) (*n* = 19) one month before being mated with male rats. During gestation, rats continued to be fed with the same diets. After delivery, litters were equated to 10 pups per dam at postnatal day (PND) 1, with 5 males and 5 females when possible. During lactation, the eight dams fed with SD continued with it (CON-dams), while those fed WD were divided in two groups: nine dams continued with WD (WD-dams), and ten dams were switched to SD (REV-dams).

Male and female pups within each litter of CON-, WD- and REV-dams (referred as O-CON, O-WD and O-REV) were supplemented with an oral solution of recombinant murine leptin (PeproTech, London, UK) (O-CON-L, O-WD-L and O-REV-L, respectively) equivalent to five times the average amount ingested from maternal milk [15], or the corresponding vehicle (water) (O-CON-V, O-WD-V and O-REV-V, respectively) during the whole suckling period, from PND1 to PND20, using a pipette. The specific daily doses for the consecutive 20 days of lactation were 1.0, 2.0, 3.0, 4.0, 5.0, 6.3, 7.5, 8.8, 10.0, 11.3, 15.6, 17.2, 18.8, 20.3, 21.9, 23.5, 25.0, 26.6, 39.4 and 43.8 ng [15]. Animals were weaned at PND21, and approximately half of each group, considering maternal diet, treatment, and sex, was fed with SD and the other half with WD (*n* = 8–12 per group). Rats were separated two by two (males) or three by three (females) with other animals of the same group, and maintained with the assigned diets until four months of age. During this period, body composition was analyzed by EchoMRI-700 (Echo Medical Systems, LLC., Houston, TX, USA) at one and four months of age. Two weeks before sacrifice (at three and a half months of life), blood samples were collected in heparinized tubes from the saphenous vein and without anesthesia under 12 h fasting conditions. At four months of age, animals were sacrificed by decapitation under *ad libitum* feeding conditions. Blood was collected from the neck in heparinized containers, and the liver and retroperitoneal white adipose tissue (rWAT) were obtained, weighed and stored at −80 °C until analysis. Body weight and food intake were followed during the whole experimental period.

### 2.2. Determination of Blood Parameters under Fed/Fasting Conditions

Circulating parameters were determined in plasma at three and a half months of age, under fasting conditions, and at four months of age, under *ad libitum* feeding conditions. Plasma was obtained from blood samples by centrifugation (1000× *g*, 10 min, 4 °C). Triglycerides (TG), non-esterified fatty acids (NEFA) and cholesterol levels were measured using the Serum Triglyceride (Sigma Diagnostics, St. Louis, MO, USA), NEFA-HR (Wako Chemicals GmbH, Neuss, Germany) and Cholesterol (Biosystems SA, Barcelona, Spain) enzymatic colorimetric kits, respectively. Enzyme-linked immunosorbent assay (ELISA) kits were used to determine insulin (Mercodia AB, Uppsala, Sweden) and leptin (R&D Systems, Minneapolis, MN, USA) levels. Glucose levels were measured in fresh blood by Accu-Check Glucometer (Roche Diagnostics, Barcelona, Spain). The homeostatic model assessment (HOMA-IR) was used to evaluate the insulin-resistant state of the animals by using the formula HOMA-IR = [fasting glucose (mmol·L^−^^1^) × fasting insulin (mU·L^−^^1^)]/22.5 [22]. 

### 2.3. Extraction and Quantification of Hepatic Lipid Content 

Total hepatic lipids were extracted from approximately 0.3 g of liver with a hexane:isopropanol mixture (3:2, *v*:*v*), as previously described [23]. After extraction, the fatty phase was dissolved in hexane and dried with nitrogen gas. The fat weight was determined and expressed relative to the amount of wet liver weight (mg·g^−^^1^) [23].

### 2.4. RNA Extraction

Total RNA was extracted from liver and rWAT of 4-month-old animals using the E.Z.N.A. Total RNA Kit I (Omega Bio-Tek, Inc., Norcross, GA, USA). After extraction, RNA was quantified using the NanoDrop ND-1000 spectrophotometer (NanoDrop Technologies, Inc., Wilmington, DE, USA) and its integrity confirmed using 1% agarose gel electrophoresis.

### 2.5. Real-Time Quantitative Polymerase Chain Reaction (RT-qPCR) Analysis

RT-qPCR was used to measure mRNA expression levels of leptin (*Lep*), leptin receptor (*Lepr*), insulin receptor (*Insr*), peroxisome proliferator-activated receptor gamma (*Pparγ*), patatin-like phospholipase domain containing 2 (*Pnpla2*) and Cd36 molecule (*Cd36*) in rWAT, and of insulin receptor substrate 1 (*Irs1*), sterol regulatory element binding transcription factor 1 (*Srebp1*), carnitine palmitoyltransferase 1 (*Cpt1*), *Insr*, stearoyl-CoA desaturase 1 (*Scd1*), peroxisome proliferator-activated receptor alpha (*Pparα*), *Lepr*, *Cd36* and sterol regulatory element binding transcription factor 2 (*Srebp2*) in liver, as previously described [9]. As housekeeping gene, guanosine diphosphate dissociation inhibitor (*Gdi*) was selected. Sequences and amplicon size of primers used (Sigma; Madrid, Spain) are detailed in Supplementary Materials Table S1.

### 2.6. Statistical Analysis

Data are represented as the mean ± SEM. First, data were separated depending on the offspring diet (OD), and differences between experimental groups were analyzed by Factorial Analysis of Variance (ANOVA), considering three factors (three-way ANOVA): sex (S), maternal diet (MD) and/or leptin treatment (L). Next, after the separation of the data by OD and S of animals, differences due to two factors (MD and/or L) were analyzed by two-way ANOVA. Finally, when an interactive effect between MD and L was found, data were also separated by L and one-way ANOVA performed to detect differences due to MD into each treatment. All ANOVA tests were followed by a least significant difference (LSD) post-hoc test when the effect of MD was significant. Single comparisons between leptin-treated groups and vehicle-treated groups were carried out by the Mann-Whitney U test. The Shapiro-Wilk and Bartlett tests were used to evaluate the normality and homogeneity of variances of the data, respectively. Analyses were carried out with SPSS for Windows (SPSS, Chicago, IL, USA), with the threshold of significance at *p* < 0.05.

## 3. Results

### 3.1. Food Intake and Body Weight-Related Parameters

**Figure 2:**
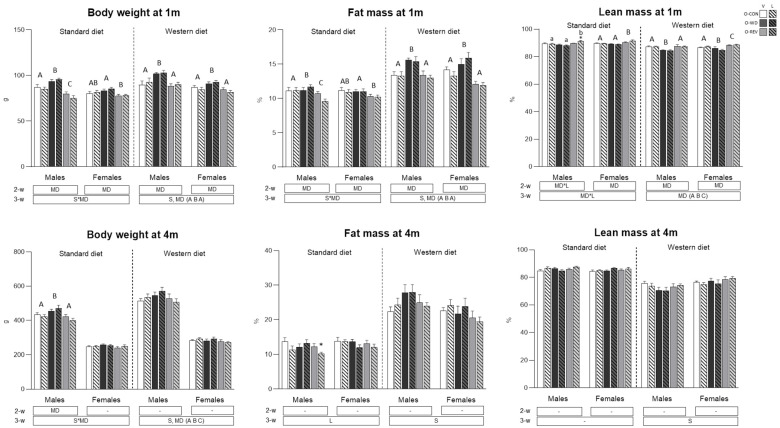
Body weight and composition (fat and lean mass percentage) of O-CON, O-WD and O-REV male and female animals treated with vehicle or leptin during suckling and fed SD or WD from weaning, at one and four months of age. Data are mean ± SEM (*n* = 8–12). Statistics: After data separation depending on post-weaning diet, three-way ANOVA was performed to analyze the effect of sex, maternal diet and/or leptin treatment. In each sex, two-way ANOVA was performed to analyze the effect of leptin treatment and/or maternal diet. When an interactive effect was found, one-way ANOVA was performed to analyze the effect of maternal diet in each treatment. Single comparisons between leptin versus vehicle-treated rats of all experimental groups were carried out using Mann-Whitney U test. Symbols: sex (S), maternal diet (MD), leptin treatment (L); A ≠ B ≠ C (*p* < 0.05, LSD post-hoc, two-way ANOVA); a ≠ b (*p* < 0.05, LSD post-hoc, one-way ANOVA); *, different from their vehicle-treated equal (*p* < 0.05, Mann-Whitney U test). Abbreviations: offspring of CON-dams (O-CON), offspring of WD-dams (O-WD), offspring of REV-dams (O-REV), vehicle (V), leptin (L).

Body weight and composition at one and four months of age of O-CON, O-WD and O-REV male and female animals, treated with vehicle or leptin during the suckling period and fed SD or WD from weaning, are shown in Figure 2. At one month of life, O-WD (only males under SD, and both males and females under WD) showed higher body weight and fat mass percentage than O-CON animals (two- and three-way ANOVA, respectively), whereas both parameters were totally normalized in O-REV rats, being in the case of male rats under SD even lower than in O-CON animals (two-way ANOVA). O-REV females under SD also showed lower body weight and fat mass percentage than O-WD animals, but both parameters were not different from O-CON animals (two-way ANOVA). Lean mass percentage in O-REV males (considering only leptin-treated groups) and females was higher than in O-CON and O-WD animals under SD feeding conditions (one- and two-way ANOVA, respectively), with O-REV-L males showing a higher value than O-REV-V males (Mann-Whitney U-test). Under WD feeding, O-WD rats (males and females) showed a lower lean mass percentage in comparison with O-CON and O-REV groups, and O-REV animals, particularly females, also displayed a higher value than O-CON animals (three-way ANOVA). At four months of life, O-WD (only males under SD, and both males and females under WD) continued displaying higher body weight, but not higher fat mass percentage, than O-CON and O-REV animals (two- and three-way ANOVA, respectively). Under WD feeding, O-REV rats showed even lower body weight than O-CON animals (three-way ANOVA). Under SD, both male and female leptin-treated groups presented lower fat mass percentage than their respective vehicle-treated groups (three-way ANOVA), especially in the case of O-REV males (Mann-Whitney U test). 

**Figure 3:**
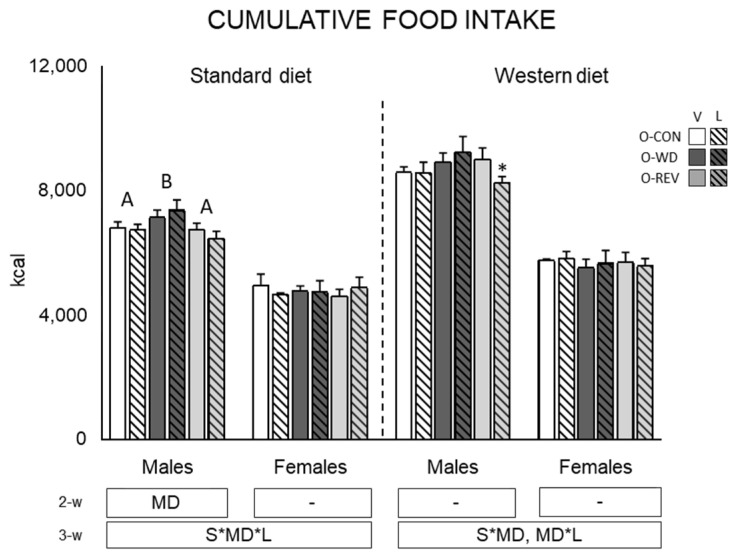
Cumulative food intake from one to four months of age of O-CON, O-WD and O-REV male and female animals treated with vehicle or leptin during suckling and fed SD or WD from weaning. Data are mean ± SEM (*n* = 8–12). Statistics: After data separation depending on post-weaning diet, three-way ANOVA was performed to analyze the effect of sex, maternal diet and/or leptin treatment. In each sex, two-way ANOVA was performed to analyze the effect of leptin treatment and/or maternal diet. Single comparisons between leptin versus vehicle-treated rats of all experimental groups were carried out using Mann-Whitney U test. Symbols: sex (S), maternal diet (MD), leptin treatment (L); A ≠ B (*p* < 0.05, LSD post-hoc, two-way ANOVA); *, different from their vehicle-treated equal (*p* < 0.05, Mann-Whitney U test). Abbreviations: offspring of CON-dams (O-CON), offspring of WD-dams (O-WD), offspring of REV-dams (O-REV), vehicle (V), leptin (L).

Figure 3 shows cumulative food intake (from one to four months of life) of O-CON, O-WD and O-REV male and female animals, treated with vehicle or leptin during the suckling period and fed SD or WD from weaning. Under SD conditions, O-WD males displayed a higher food intake than O-CON and O-REV animals (two-way ANOVA). A similar trend was observed under WD conditions, but results did not reach statistical significance (*p* = 0.056, two-way ANOVA). In addition, under WD conditions, O-REV-L males presented significantly lower food intake than their respective vehicle-treated group (Mann Whitney U test).

### 3.2. Circulating Parameters

**Figure 4:**
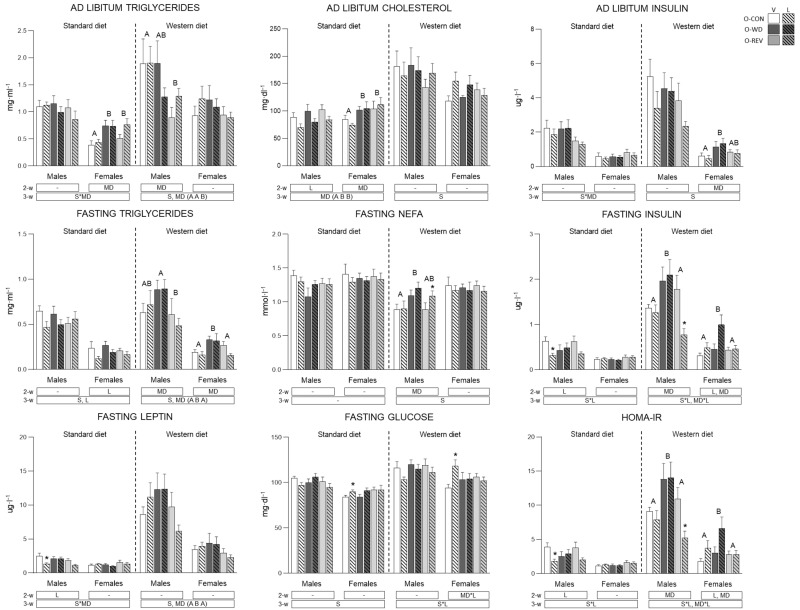
Circulating parameters and HOMA-IR of O-CON, O-WD and O-REV male and female animals treated with vehicle or leptin during suckling and fed SD or WD from weaning, under fasting (at three and half months) and ad libitum (four months) feeding conditions. Data are mean ± SEM (*n* = 8–12). Statistics: After data separation depending on post-weaning diet, three-way ANOVA was performed to analyze the effect of sex, maternal diet and/or leptin treatment. In each sex, two-way ANOVA was performed to analyze the effect of leptin treatment and/or maternal diet. Single comparisons between leptin versus vehicle-treated rats of all experimental groups were carried out using Mann-Whitney U test. Symbols: sex (S), maternal diet (MD), leptin treatment (L); A ≠ B (*p* < 0.05, LSD post-hoc, two-way ANOVA); *, different from their vehicle-treated equal (*p* < 0.05, Mann-Whitney U test). Abbreviations: offspring of CON-dams (O-CON), offspring of WD-dams (O-WD), offspring of REV-dams (O-REV), vehicle (V), leptin (L).

Circulating parameters under *ad libitum* and/or fasting conditions, together with HOMA-IR, of O-CON, O-WD and O-REV male and female animals, treated with vehicle or leptin during the suckling period, and fed SD or WD from weaning, are shown in Figure 4. 

Under SD, O-WD and O-REV showed higher levels of TG (only females, two-way ANOVA) and cholesterol (males and females, three-way ANOVA) under *ad libitum* conditions than their controls. Under WD conditions, O-WD animals displayed increased fasting TG, insulin and leptin levels, and a higher HOMA-IR than O-CON animals (two- or three-way ANOVA). O-WD males also showed higher fasting NEFA levels than their controls (two-way ANOVA). The levels of these parameters were normalized in O-REV animals, which even showed lower triglyceride levels under *ad libitum* conditions than O-CON and O-WD animals. 

Regarding the effects of leptin supplementation during suckling, it is noteworthy that, under SD feeding conditions, leptin-treated animals displayed lower fasting triglyceride levels (both males and females) (three-way ANOVA), and, in the case of males, also lower cholesterol (under *ad libitum* conditions) and fasting leptin and insulin levels, as well as reduced HOMA-IR (two-way ANOVA) compared to their respective vehicle-treated groups. Notably, the decrease of fasting plasma leptin and insulin levels and of HOMA-IR was more evident and significant (Mann-Whitney U test) in O-CON animals, whereas leptin-treated O-WD rats displayed values similar to those treated with the vehicle. O-CON-L females also showed higher fasting glucose levels than their respective vehicle-treated controls, under both SD and WD conditions (Mann-Whitney U test), and, under WD conditions, leptin-treated females showed greater fasting insulin levels and HOMA-IR than their respective vehicle-treated controls (two-way ANOVA). The increment was more apparent in O-CON and O-WD females, while the values of these parameters in O-REV females treated with leptin or vehicle were similar. Of note, under WD conditions, O-REV-L males displayed lower fasting insulin levels and HOMA-IR and higher fasting NEFA levels than O-REV-V (Mann-Whitney U test).

### 3.3. Hepatic Lipid Content

**Figure 5:**
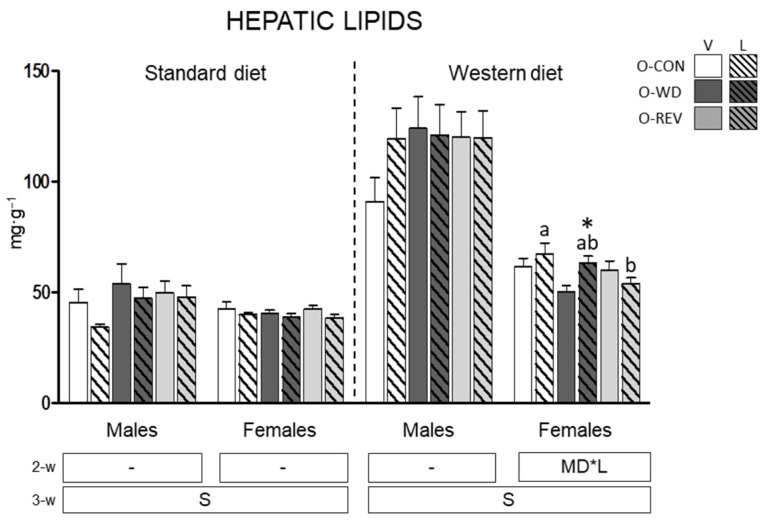
Hepatic lipid content in O-CON, O-WD and O-REV male and female animals treated with vehicle or leptin during suckling and fed SD or WD from weaning, at four months of age. Data are mean ± SEM (*n* = 8–12). Statistics: After data separation depending on post-weaning diet, three-way ANOVA was performed to analyze the effect of sex, maternal diet and/or leptin treatment. In each sex, two-way ANOVA was performed to analyze the effect of leptin treatment and/or maternal diet. When an interactive effect was found, one-way ANOVA was performed to analyze the effect of maternal diet in each treatment. Single comparisons between leptin versus vehicle-treated rats of all experimental groups were carried out using Mann-Whitney U test. Symbols: sex (S), maternal diet (MD), leptin treatment (L); a ≠ b (*p* < 0.05, LSD post-hoc, one-way ANOVA); *, different from their vehicle-treated equal (*p* < 0.05, Mann-Whitney U test). Abbreviations: offspring of CON-dams (O-CON), offspring of WD-dams (O-WD), offspring of REV-dams (O-REV), vehicle (V), leptin (L).

Total hepatic lipids of O-CON, O-WD and O-REV male and female animals, treated with vehicle or leptin during suckling, and fed SD or WD from weaning, are presented in Figure 5. Under WD feeding, leptin-treated O-REV females showed lower hepatic lipid content than O-CON rats (one-way ANOVA), whereas a significant increase was observed in O-WD-L females in comparison with their respective vehicle-treated group (Mann-Whitney U test).

### 3.4. Expression of Energy Metabolism-Related Genes in rWAT and Liver

**Figure 6:**
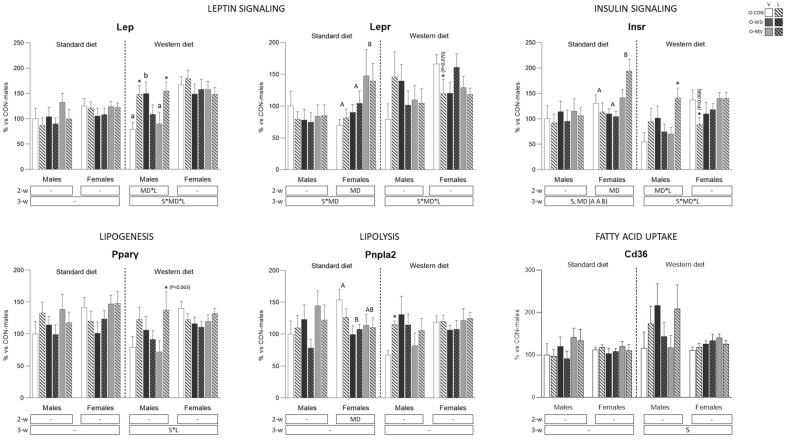
Expression levels of genes related to energy metabolism in retroperitoneal white adipose tissue of O-CON, O-WD and O-REV male and female animals treated with vehicle or leptin during suckling and fed SD or WD from weaning, at four months of age. Data are mean ± SEM (*n* = 8–12). Statistics: After data separation depending on post-weaning diet, three-way ANOVA was performed to analyze the effect of sex, maternal diet and/or leptin treatment. In each sex, two-way ANOVA was performed to analyze the effect of leptin treatment and/or maternal diet. When an interactive effect was found, one-way ANOVA was performed to analyze the effect of maternal diet in each treatment. Single comparisons between leptin versus vehicle-treated rats of all experimental groups were carried out using Mann-Whitney U test. Symbols: sex (S), maternal diet (MD), leptin treatment (L); A ≠ B (*p* < 0.05, LSD post-hoc, two-way ANOVA); a ≠ b (*p* < 0.05, LSD post-hoc, one-way ANOVA); *, different from their vehicle-treated equal (*p* < 0.05, Mann-Whitney U test). Abbreviations: offspring of CON-dams (O-CON), offspring of WD-dams (O-WD), offspring of REV-dams (O-REV), vehicle (V), leptin (L).

Figure 6 shows expression levels of energy metabolism-related genes in rWAT. Under SD conditions, O-REV animals displayed higher mRNA expression levels of *Lepr* (only females) and *Insr* (both, but mainly females) than both O-CON and O-WD groups (two- and three-way ANOVA). O-WD females showed lower *Pnpla2* expression than O-CON animals, and expression levels in O-REV females were not different from both O-CON and O-WD animals (two-way ANOVA). Under WD conditions, O-WD-V male rats showed higher *Lep* expression levels than O-CON-V and O-REV-V (one-way ANOVA), whereas O-CON and O-REV males that were treated with leptin showed higher expression levels than their respective vehicle-treated controls (Mann-Whitney U test). Also in males, and under WD conditions, leptin treatment also resulted in increased expression levels of *Pnpla2* in O-CON animals, and of *Insr* in O-REV animals (Mann-Whitney U test). 

**Figure 7:**
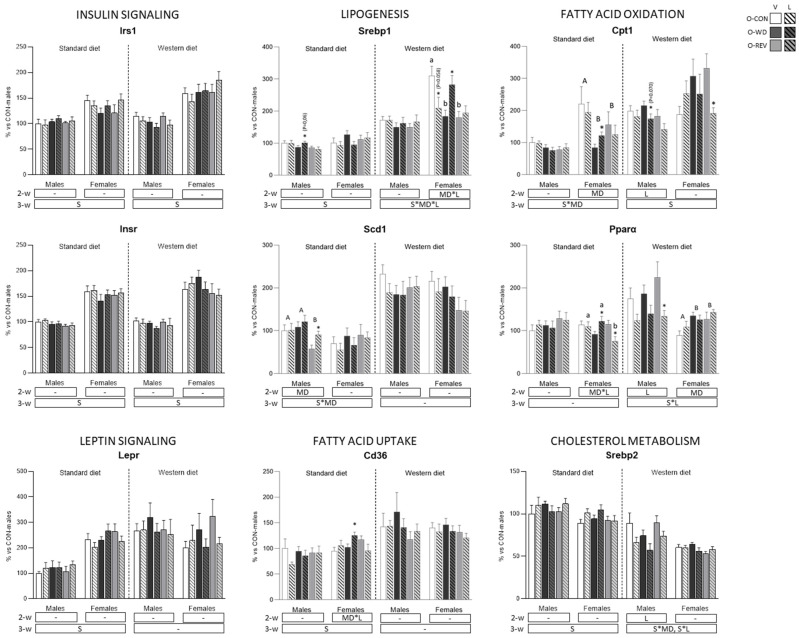
Expression levels of genes related to energy metabolism in liver of O-CON, O-WD and O-REV male and female animals treated with vehicle or leptin during suckling and fed SD or WD from weaning, at four months of age. Data are mean ± SEM (*n* = 8–12). Statistics: After data separation depending on post-weaning diet, three-way ANOVA was performed to analyze the effect of sex, maternal diet and/or leptin treatment. In each sex, two-way ANOVA was performed to analyze the effect of leptin treatment and/or maternal diet. When an interactive effect was found, one-way ANOVA was performed to analyze the effect of maternal diet in each treatment. Single comparisons between leptin versus vehicle-treated rats of all experimental groups were carried out using Mann-Whitney U test. Symbols: sex (S), maternal diet (MD), leptin treatment (L); A ≠ B (*p* < 0.05, LSD post-hoc, two-way ANOVA); a ≠ b (*p* < 0.05, LSD post-hoc, one-way ANOVA); *, different from their vehicle-treated equal (*p* < 0.05, Mann-Whitney U test). Abbreviations: offspring of CON-dams (O-CON), offspring of WD-dams (O-WD), offspring of REV-dams (O-REV), vehicle (V), leptin (L).

Figure 7 shows expression levels of energy metabolism-related genes in liver. Under SD conditions, O-REV males displayed lower mRNA expression levels of *Scd1* than O-CON and O-WD rats, and O-WD and O-REV females showed lower *Cpt1* mRNA levels than controls (two-way ANOVA). Notably, in males, leptin treatment resulted in increased expression levels of *Scd1* in O-REV and of *Cpt1* in O-WD rats (Mann-Whitney U test). In addition, also under SD conditions, the effect of leptin treatment on gene expression in females was different depending on maternal conditions, since it resulted in increased mRNA levels of *Pparα* and *Cd36* in O-WD and decreased expression levels of *Pparα* in O-REV (Mann-Whitney U test). Thus, expression levels of *Pparα* in O-REV-L females were significantly lower than those of O-CON-L and O-WD-L (one-way ANOVA). Under WD conditions, O-WD and O-REV females presented decreased *Srebp1* (only vehicle-treated rats) and increased *Pparα* expression levels than their control (one- and two-way ANOVA, respectively). Under these conditions, in females, leptin treatment resulted in lower expression levels of *Cpt1* in O-REV rats and higher expression levels of *Srebp1* in O-WD (Mann-Whitney U test); in males, leptin treatment resulted in lower expression levels of *Cpt1*, *Pparα* (especially O-REV-L rats, Mann-Whitney U test) and *Srebp2* (two-way ANOVA).

## 4. Discussion

The present study has evaluated the effects of leptin supplementation during the suckling period in the male and female offspring of diet-induced obese dams due to exposure to an obesogenic diet before and during pregnancy and lactation, as well as its effects when implementing a healthier maternal diet during the lactation period. In addition, since the consequences of leptin treatment in suckling rats may also respond to nutritional insults later in life [15,17,20], the influence of WD exposure in post-weaning rats has also been studied. 

Maternal obesity and obesogenic diets during gestation and lactation may have a negative impact on the programming of later metabolic health of the offspring and have been associated with an increased risk of obesity [12]. In this regard, we have previously shown that implementation of a healthy diet during lactation in diet-induced obese dams attenuates detrimental programming effects in their offspring observed at an early age [9]. This was associated with the normalization of milk composition [9,10]. Here, in the same cohort studied at early age [9], it is also highlighted that maternal intake of an obesogenic diet before and during gestation and lactation has detrimental effects on the phenotype of the adult offspring, and that these effects are magnified by exposure to WD conditions after weaning. Other studies have also described that the negative outcomes associated with maternal overnutrition during the perinatal period on the offspring are exacerbated when animals are exposed to unbalanced diets later in life [24,25]. Interestingly, here it is shown that most of the adverse effects in the adult offspring were prevented or attenuated by adequate maternal nutrition during lactation. Specifically, the offspring of diet-induced obese dams had a higher body weight and fat mass percentage, more prominent at an earlier age, with the exception of female rats under SD. The greater body weight in the male offspring of WD-dams under SD conditions is in agreement with the higher cumulative food intake observed in these animals. Moreover, the offspring of WD-dams displayed alterations in circulating parameters under WD conditions, i.e., increased fasting TG, leptin and insulin levels, and greater HOMA-IR than their controls, along with increased fasting NEFA levels (only males) and *ad libitum* insulin levels (only females). These alterations were not found in the offspring of obese dams with dietary normalization during lactation (REV-dams). In addition, under WD conditions, the female offspring of REV-dams that were treated with leptin even displayed lower hepatic lipid content than the offspring of CON-dams. However, other alterations found in the offspring of WD-dams, under SD conditions, such as increased *ad libitum* TG (females) and cholesterol (males and females), in comparison to their controls, were not normalized by dietary improvement during lactation. 

Specific milk components may play a relevant role in the growth and development of the neonate, imprinting future protection against chronic diseases [26,27]. In this sense, a critical role of leptin during lactation in the protection against the development of obesity-related metabolic alterations in adulthood has been demonstrated, particularly in animal models [13]. Oral supplementation with physiological doses of leptin during the suckling period has been shown to improve later metabolic health in male rats, both in the offspring of control rats [15,16,17] and of rats with moderate gestational calorie restriction [18,19,20]. Moreover, such studies have evidenced that leptin supplementation also improves the response to an obesogenic challenge in the adult offspring, either a high-fat diet in the offspring of well-nourished dams [15,17] or a WD in the offspring of calorie-restricted dams during gestation [20]. However, to the best of our knowledge, whether leptin can exert these protective effects in the offspring of rats with diet-induced obesity, exposed or not to an obesogenic diet during lactation, has not been studied so far.

Here, it is shown that leptin treatment during lactation had no apparent effect on body weight in none of the experimental groups studied, at least until four months of age. It should be noted that, in previous studies, the effects on body weight have been shown in adulthood both from an earlier age (three months of age) [15], and later, from six months, approx. [16]. However, despite no differences in body weight, leptin treatment resulted in lower body fat mass percentage in animals that were weaned on a SD. This effect was especially marked in the male offspring of REV-dams, which also displayed lower food intake when exposed to WD. Moreover, leptin treatment also resulted in lower fasting TG (in males and females), leptin and insulin levels, *ad libitum* cholesterol levels and HOMA-IR (in males) under SD conditions. Interestingly, the effects of leptin on fasting leptin and insulin levels and HOMA-IR were more marked in the male offspring of CON-dams, while the effects on the offspring of WD-dams, in particular, were not appreciable. Moreover, leptin supplementation also resulted in lower fasting insulin levels and HOMA-IR in the male offspring of REV-dams under WD, but it did not prevent or attenuate the marked increase in these parameters observed in the offspring of WD-dams. Thus, these results suggest that the metabolic malprogramming caused by maternal WD consumption throughout the perinatal period, and manifested mainly when pups are exposed to obesogenic conditions from weaning, cannot be reversed by leptin supplementation during the suckling period. Furthermore, in the case of females exposed from weaning to WD conditions, even the opposite effect was observed. Fasting insulin levels and HOMA-IR were increased in female rats that were supplemented with leptin, with the most notable effects in the offspring of WD-dams (increases of 118% and 122%, respectively), while said increases were almost negligible in the offspring of REV-dams (increases of 5.3% and 2.2%, respectively). In addition, the female offspring of WD-dams that were treated with leptin and weaned on a WD also showed increased hepatic lipid content, in comparison with their vehicle-treated controls that showed decreased values than the offspring of CON-dams. The lack of effect or even some apparent adverse effects of leptin in the female offspring of WD-dams do not appear to be due to excess leptin intake during lactation, as the concentration of leptin in the milk of their mothers was even lower than that of their controls on day 15 of lactation [9], although both males and females did present hyperleptinemia throughout lactation due to their greater adiposity [9]. It could be speculated that changes in milk composition in WD-dams could interfere with leptin action during this critical period of development. Of note, the implementation of a healthy diet during lactation was shown to reverse most of the alterations in milk composition [9,10]. This could explain the improvement of the metabolic programming of the offspring, and in turn the recovery of the beneficial effects of oral leptin during the suckling period, which were observed particularly in males. This is consistent with previous studies showing that the offspring of post-cafeteria rats did not present the expected negative outcomes of maternal overweight in terms of excess adiposity and metabolic alterations [28]. This was tentatively related to the intake of higher amounts of leptin from maternal milk during the suckling period (due to greater maternal adiposity) in the absence of alterations in maternal diet. Taken together, these results would be in accordance with a greater influence of maternal diet on milk composition and on offspring metabolic programming, rather than maternal obesity per se, as previously proposed [9,10]. 

**Figure 8:**
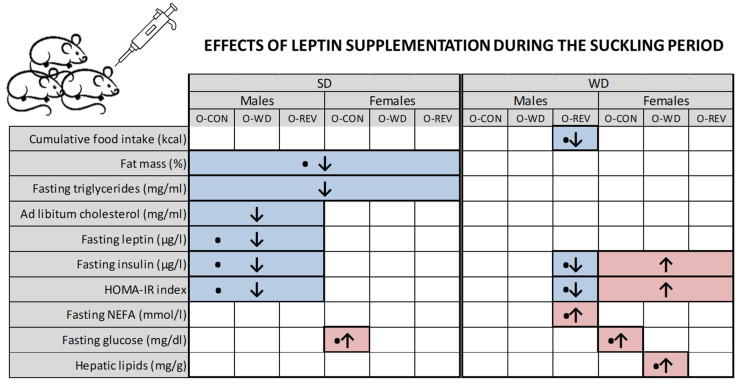
Summary of the effects of leptin supplementation during suckling on cumulative food intake (from one to four months of age), fat mass and hepatic lipid content (four months), and circulating parameters in adult O-CON, O-WD and O-REV male and female rats weaned onto SD or WD. Circulating parameter were determined at four months (under feeding conditions), or at three and a half months (under fasting conditions). Abbreviations: offspring of CON-dams (O-CON), offspring of WD-dams (O-WD), offspring of REV-dams (O-REV), standard diet (SD), western diet (WD). Symbols: ↑ and ↓ indicate increases or decreases, respectively, by Mann-Whitney U test, two-way ANOVA, or three-way ANOVA with respect to their vehicle-treated equals; ●, significant difference in the indicated group with respect to its vehicle-treated equal by Mann-Whitney U test.

It is worth mentioning that the long-term effects of leptin supplementation during the suckling period have so far been studied mainly in male animals [13]. Of interest, here it is shown that the beneficial effects of leptin are certainly more evident in males than in females, regardless of the experimental group, at least for the parameters studied. This could be related to the fact that females are more protected against metabolic alterations in adulthood [29,30,31], but we cannot rule out other possible causes related to differences between the sexes. Moreover, we show here some negative response to leptin treatment in females, even in the control group. For example, the female offspring of CON-dams treated with leptin displayed increased fasting glucose levels, both when exposed to SD and WD conditions, compared with their vehicle-treated controls. On the other hand, the effects are also more noticeable in the offspring of CON- and REV-dams, rather than in the offspring of WD-dams (see a summary of the main long-term effects of leptin supplementation during the suckling period in Figure 8).

The analysis of the expression levels of key energy metabolism-related genes in WAT and liver gives some clues to the programming effects by maternal conditions and leptin supplementation during the suckling period. Regarding WAT, the male offspring of CON- and REV-dams treated with leptin and exposed post-weaning to WD conditions showed increased *Lep* gene expression compared to the vehicle-treated groups, suggesting a better response to the dietary stress. In addition, the male offspring of REV-dams also displayed increased *Insr* mRNA levels and a trend (*p* = 0.063) to higher *Pparγ* mRNA levels. Insulin plays a critical adipogenic role in the adipose tissue. On the one hand, by stimulating glucose and free fatty acid uptake, and de novo fatty acid synthesis; and on the other hand, by inhibiting lipolysis [32]. In addition, insulin also regulates adipose tissue development and differentiation by raising the gene expression of diverse fat-specific transcription factors, including PPARγ [33]. Thus, such changes suggest that O-REV males that were treated with leptin during the suckling period present increased peripheral insulin signaling, which is consistent with the presence of decreased fasting insulin levels and HOMA-IR with respect to their vehicle-treated controls. Moreover, the male offspring of CON-dams that were treated with leptin and were exposed after weaning to WD conditions also displayed higher expression levels of *Pnpla2* than their vehicle-treated controls. *Pnpla2* encodes for the enzyme adipose triglyceride lipase (ATGL), which is key in the beginning of triglyceride lipolysis [34]. These results may reflect the beneficial effect of leptin supplementation during lactation by increasing lipolysis capacity, which may be mediated, at least in part, by increased leptin action in this tissue. It is also noticeable that O-REV animals under SD conditions, treated or not with leptin, displayed increased mRNA levels of the *Lepr* (females) and *Insr* (both sexes) genes in the rWAT in comparison to both O-CON and O-WD rats, in accordance with the beneficial effects of dietary normalization during lactation. 

Gene expression analysis in liver also elucidated certain beneficial effects of leptin treatment during lactation in males. Concretely, mRNA levels of *Srebp2*, the master regulator of cholesterol biosynthesis [35], were reduced in all groups of WD-fed male rats that were supplemented with leptin during the suckling period, suggesting a better capacity to regulate plasma cholesterol levels. In fact, under SD, leptin-treated rats displayed lower plasma cholesterol levels in comparison with their vehicle-treated controls. Moreover, the presence of decreased hepatic mRNA levels of *Pparα* and *Cpt1a* in leptin-treated male rats exposed to WD conditions, irrespective of maternal diet, could be related to a greater capacity of these animals to channel excess energy supplied from the diet towards WAT, preventing ectopic fat accumulation in other organs, such as the liver. This suggests a better adaptive response to a hypercaloric diet and may be related to increased insulin sensitivity [29]. Conversely, the presence of increased expression levels of *Srebp1* due to leptin treatment in the female offspring of WD-dams and exposed to WD conditions, which was not found in the offspring of CON- or REV-dams, suggests increased hepatic lipogenesis and is consistent with the increased hepatic lipid content found in these animals. These alterations may be related to an impairment in insulin sensitivity, as suggested by the notable increase in fasting insulin levels and the higher HOMA-IR. However, it must be mentioned that *Srebp1* mRNA levels in liver in the female offspring of WD-dams that were treated with the vehicle and weaned on a WD were lower than levels in the offspring of CON-dams, suggesting that the adaptations programmed in the liver by the maternal diet would allow them to better cope with an obesogenic environment. However, it remains to be determined whether these apparent advantages actually translate into an improved phenotype at older ages.

## 5. Conclusions

The programming effects of leptin ingested during the suckling period depend on maternal dietary conditions and the diet followed from weaning, besides the sex of animals. Particularly, leptin supplementation during the suckling period seems to improve metabolic health in adult males more strongly than in females, especially evident under SD. Maternal WD throughout the perinatal period coupled with a subsequent obesogenic diet in offspring weakened this beneficial effect of leptin, even resulting in impaired insulin sensitivity in females. In contrast, switching to a healthy maternal diet during lactation, despite maintaining maternal obesity, was able to offset such reduction in the effects of leptin, particularly in males. All in all, these findings reflect the strong impact of maternal diet during lactation, rather than the obese status itself, on the future metabolic health of the offspring, as well as on the beneficial effects of leptin supplemented at physiological doses during the suckling period, which are more evident in the offspring (particularly males) of dams on a balanced diet during lactation.

## Data Availability

The data that support the findings of this study are available from the corresponding author upon reasonable request.

## Supplementary Materials

The following supporting information can be downloaded at: https://www.mdpi.com/article/10.3390/nu15030570/s1, Table S1: Nucleotide sequences and amplicon size of primers used for PCR amplification.
