# Supplementary material for: Influence of Maternal Metabolic Status and Diet during the Perinatal Period on the Metabolic Programming by Leptin Ingested during the Suckling Period in Rats
Source: Nutrients. 2023 Jan 21;15(3):570. doi: 10.3390/nu15030570 (PMC9921535; doi:10.3390/nu15030570)
Supplement: Supplementary file 1 [file nutrients-15-00570-s001.zip › nutrients-2100982-supplementary.pdf]

Supplementary Materials

Table S1. Nucleotide sequences and amplicon size of primers used for PCR amplification

Gene	Forward primer (5' to 3')	Reverse primer (5' to 3')	Amplicon size (pb)
Gdi	CCGCACAAGGCAAATACATC	GACTCTCTGAACCGTCATCAA	210
Irs1	GCAACCGCAAAGGAAATG	ACCACCGCTCTCAACAGG	293
Insr	GTCCGGCGTTCATCAGAG	CTCCTGGGATTCATGCTGTT	242
Srebp1	CCCACCCCCTTACACACC	GCCTGCGGTCTTCATTGT	198
Scd1	ATCCCCTCCTCCAAGGTCTA	CGGGCCCATTCATATACATC	188
Cpt1	CGAGAAGGGAGGACAGAGAC	GGACACCACATAGAGGCAGAA	201
Pparα	TGTCGAATATGTGGGGACAA	AAACGGATTGCATTGTGTGA	215
Lepr	AGCCAAACAAAAGCACCATT	TCCTGAGCCATCCAGTCTCT	174
Cd36	AATCCTCTCCCTCTCTGGTG	TAGGCAGCATGGAACTTGAC	175
Srebp2	GTCCTCACCTTCCTGGGTCT	CAGCAGTAGAGTCGGCATCA	168
Lep	TTCACACACGCAGTCGGTAT	AGGTCTCGCAGGTTCTCCAG	186
Pparγ	GATCCTCCTGTTGACCCAGA	TCAAAGGAATGGGAGTGGTC	164
Pnpla2	TGTGGCCTCATTCCTCCTAC	AGCCCTGTTTGCACATCTCT	271
